# Impact of Beta-Thalassemia Trait on Clinical Outcomes and Treatment Response in Chronic Myeloid Leukemia

**DOI:** 10.7759/cureus.102005

**Published:** 2026-01-21

**Authors:** Aytan Shirinova, Chingiz Asadov, Aypara Hasanova, Zohra Alimirzoyeva

**Affiliations:** 1 Hematology, National Hematology and Blood Transfusion Center, Baku, AZE; 2 Genetics, National Hematology and Blood Transfusion Center, Baku, AZE

**Keywords:** beta-thalassemia, blood transfusion, chronic myeloid leukemia, epidemiology, molecular response, risk assessment, treatment outcome, tyrosine kinase inhibitor

## Abstract

Background and aim

The coexistence of beta-thalassemia trait and chronic myeloid leukemia (CML) is rare, and the clinical implications of this overlap remain poorly defined. Hematologic alterations associated with beta-thalassemia trait, such as microcytosis, elevated RBC counts, and compensatory marrow activity, may influence CML diagnosis, prognostic scoring, and treatment response. Understanding these interactions is clinically important for accurate risk stratification and individualized treatment planning. This study aimed to determine whether beta-thalassemia trait affects baseline clinical characteristics, tyrosine kinase inhibitor (TKI) treatment response, transfusion requirements, and survival outcomes in patients with chronic myeloid leukemia. Additionally, this study aimed to evaluate whether thalassemia-associated hematologic features influence established prognostic scoring systems.

Methods

This retrospective cohort study included patients diagnosed with CML who were treated at the National Hematology and Blood Transfusion Center in Baku, Azerbaijan. Patients were stratified by confirmed beta-thalassemia trait status. Baseline laboratory parameters, prognostic risk categories (Sokal, Hasford, European Treatment and Outcome Study {EUTOS} score), molecular response milestones, transfusion frequency, treatment modifications, and survival outcomes were compared. Statistical analyses included chi-square tests, Fisher’s exact tests, independent t-tests, and Kaplan-Meier survival analysis. Effect estimates were reported with p-values and absolute differences where relevant. Ethics approval or waiver was obtained from the institutional review board.

Results

Among 848 CML patients, 15 (1.8%) had beta-thalassemia trait; 30 age- and sex-matched non-trait controls were selected. Trait prevalence was significantly lower than expected from the national background rate (p=0.00018). Trait carriers showed lower mean corpuscular volume (p<0.001), higher platelet counts (p=0.045), and more frequent Sokal low-risk classification (p=0.041). Time to major molecular response did not differ significantly (15.3 vs. 16.3 months; p=0.291). TKI switching was less common among trait carriers (46.7% vs. 66.7%; p=0.218). Transfusion needs were higher in the trait group (40% vs. 13.3%; p=0.099). No statistically significant differences were found in progression or survival outcomes.

Conclusions and relevance

Beta-thalassemia trait influences baseline hematologic profiles and risk stratification in CML but does not impair the effectiveness of tyrosine kinase inhibitors or survival. The higher transfusion rates suggest the need for closer anemia monitoring. Additional multicenter research is warranted to guide personalized management in CML patients with underlying hemoglobinopathies.

## Introduction

Chronic myeloid leukemia (CML) is a clonal myeloproliferative disorder characterized by the Philadelphia chromosome, a reciprocal translocation between chromosomes 9 and 22 that creates the BCR-ABL1 fusion oncogene. This constitutively active tyrosine kinase drives unchecked proliferation of myeloid precursors and represents both the diagnostic hallmark and therapeutic target of CML [1]. The advent of tyrosine kinase inhibitors (TKIs) has revolutionized CML management, transforming a once-lethal disease into a manageable chronic condition for most patients [2,3].

Beta-thalassemia trait, or beta-thalassemia minor, is a widespread hereditary hemoglobinopathy marked by diminished beta-globin chain production. While individuals with the trait are typically asymptomatic or only mildly anemic, the condition is associated with microcytosis, elevated HbA2 levels, and sometimes splenomegaly [4,5]. These hematologic alterations can complicate the interpretation of blood parameters and disease staging in patients with coexisting hematologic malignancies. In regions with high carrier prevalence, such as the Mediterranean, Middle East, and parts of Asia, the coexistence of beta-thalassemia trait and malignancies such as CML, although uncommon, is clinically relevant [6].

To address this public health challenge, Azerbaijan has implemented a national thalassemia prevention program including premarital carrier screening and prenatal diagnosis since 2015, leading to a significant reduction in the birth rate of affected fetuses (approximately 63 fetuses diagnosed as affected among 271 fetal samples and high uptake of elective termination among affected pregnancies) and increasing public awareness of carrier status through systematic programs [7,8]. Despite this progress, the interaction between the beta-thalassemia trait and CML has not been systematically evaluated.

Despite the global burden of both conditions, the interaction between beta-thalassemia trait and CML remains poorly understood. The available literature is limited to isolated case reports and small series, and no large-scale studies have systematically examined whether beta-thalassemia trait affects CML’s clinical trajectory, risk stratification, treatment response, or survival outcomes [9]. Several theoretical interactions have been proposed as follows: baseline anemia and microcytosis in thalassemia trait may confound CML risk scores, such as the Sokal score (which incorporates age, spleen size, platelet count, and peripheral blood blast percentage), the Hasford (Euro) score (which includes age, spleen size, platelet count, eosinophils, basophils, and blast percentage), and the European Treatment and Outcome Study (EUTOS) score (based on spleen size and basophil percentage), while splenomegaly might be mistaken for leukemic infiltration, and altered erythropoiesis could influence drug metabolism or response dynamics. Furthermore, the risk of transfusion dependence during TKI therapy may be greater in trait carriers due to lower hematologic reserve [10].

To bridge this knowledge gap, we conducted a retrospective cohort study to compare the clinical features, treatment responses, and outcomes of CML patients with and without beta-thalassemia trait. We aimed to evaluate whether the coexistence of a beta-thalassemia trait influences risk-scoring accuracy, TKI tolerance, molecular response, transfusion needs, and long-term survival. Through a detailed analysis of these factors in a real-world cohort, our study aimed to shed light on the interaction between inherited hemoglobinopathies and acquired myeloid neoplasms, potentially guiding improved disease monitoring and personalized treatment strategies.

## Materials and methods

This retrospective cohort study was conducted at the National Hematology and Blood Transfusion Center of Azerbaijan and included adult patients diagnosed with chronic myeloid leukemia (CML) between January 2013 and December 2024 (a 12-year study period). The medical records of all 848 CML patients were analyzed. As the exclusive national referral center, our registry encompasses nearly all diagnosed CML cases in the country, making this dataset nationally representative.

Group assignment and diagnostic assessment

For comparison of the clinical course and outcomes between CML patients with beta-thalassemia trait and those without it, a control group of 30 patients was randomly selected from the non-trait population. The control group was matched to the trait group by sex and age using categorical matching to ensure comparability between cohorts. Matching was performed using Python (pandas library) to ensure demographic comparability between groups [11]. Beta-thalassemia trait was confirmed through hemoglobin electrophoresis or high-performance liquid chromatography (HPLC), with an HbA2 level ≥3.5% as the diagnostic threshold [4]. Only patients with beta-thalassemia trait were included in the trait group (group A), while patients with normal hemoglobin profiles constituted the control group (group B).

Data collection

Clinical and laboratory data were collected through retrospective chart review and included demographic parameters (age, sex, and region), baseline hematologic indices (hemoglobin level, mean corpuscular volume {MCV}, white blood cell {WBC} count, and platelet count), and clinical features, such as spleen size measured in centimeters below the costal margin during physical examination. Prognostic risk scores at diagnosis, including the Sokal score (which incorporates age, spleen size, platelet count, and peripheral blood blast percentage), the Hasford (Euro) score (which includes age, spleen size, platelet count, eosinophil and basophil percentages, and blast count), and the EUTOS score (based primarily on spleen size and basophil percentage), were recorded for all patients [12]. Treatment data encompassed the initial tyrosine kinase inhibitor (TKI) prescribed, dose at initiation, subsequent dose modifications, and any switches to alternative TKIs [2,3].

Supportive care measures were assessed, including the need for red blood cell transfusions during the course of TKI therapy. Transfusion dependency was defined as receiving one or more transfusions post-CML diagnosis. Additionally, the frequency of transfusions, TKI dose modifications, and associated cytopenias were evaluated to explore tolerability in trait vs. non-trait patients.

Therapeutic response was assessed by documenting the dates of complete hematologic response (CHR) and major molecular response (MMR). CHR was defined as normalization of peripheral blood counts and resolution of splenomegaly, while MMR was defined as a BCR-ABL1 transcript level ≤0.1% on the International Scale (IS) [1,3]. Additional parameters collected included disease progression to the accelerated or blast phase, death from any cause, and duration of follow-up in months.

Statistical analysis

Statistical analysis was performed using SPSS version 25.0 (Armonk, NY: IBM Corp.) and Python (Wilmington, DE: Python Software Foundation) [11,13]. Continuous variables were compared using either independent-sample t-tests or Mann-Whitney U tests, depending on distribution normality [14]. Fisher’s exact test was used to compare the frequency of beta-thalassemia trait among CML patients with its general prevalence in the population [14]. Categorical variables, such as risk score categories, transfusion requirement, dose changes, and treatment switching, were analyzed using the chi-square test [14]. Time-to-event outcomes, including progression-free survival (PFS) and overall survival (OS), were evaluated using Kaplan-Meier survival curves, with differences between groups compared using the log-rank test [14]. Median survival times were estimated where applicable. A p-value less than 0.05 was considered indicative of statistical significance.

Ethical considerations

The study protocol was reviewed and approved by the Institutional Ethics Committee of the National Hematology and Blood Transfusion Center, and all procedures were conducted in accordance with the ethical standards of the 1964 Declaration of Helsinki and its later amendments [15]. As this study was retrospective in nature and involved no direct patient contact or intervention, the requirement for informed consent was waived by the committee.

## Results

Patient demographics

A total of 45 adult patients diagnosed with chronic myeloid leukemia (CML) were included in the analysis. Among them, 15 patients (33.3%) had coexisting beta-thalassemia trait (group A), and 30 (66.7%) had no known hemoglobinopathy (group B). The median age at diagnosis was 35.3 years (range: 21-50 years) in the thalassemia trait group and 38.6 years (range: 22-62 years) in the non-trait group; the difference was not statistically significant (p=0.354). There was no significant gender imbalance between the groups.

A comparison of thalassemia trait prevalence between the CML cohort and the general population revealed a statistically significant difference. While approximately 4% of the general population in Azerbaijan carries beta-thalassemia trait, only 1.77% (15 out of 848) of CML patients were trait carriers [7,8]. This inverse association was statistically significant (Fisher’s exact test, p=0.0005), corresponding to an odds ratio of 0.43 (95% confidence interval: 0.26-0.73). These findings suggest a potential protective association between beta-thalassemia trait and the development of CML.

Clinical characteristics

Hematologic parameters at diagnosis showed distinct differences consistent with the presence of thalassemia trait. Mean corpuscular volume (MCV) was significantly lower in patients with beta-thalassemia trait (63.6 fL vs. 86.2 fL; p<0.0001), as expected due to microcytosis. Although hemoglobin levels were slightly lower in the trait group (89.4 vs. 91.4 g/L), the difference was not statistically significant (p=0.962). Platelet counts were significantly elevated in the thalassemia trait group compared to the non-trait group (mean: 687×10^9^/L vs. 331×10^9^/L; p=0.0003), while total white blood cell (WBC) counts showed no significant difference (116.5×10^9^/L vs. 125.4×10^9^/L, p=0.981). Spleen size measurements (in cm below the costal margin) did not differ significantly, although baseline splenomegaly in the trait group may be partially attributable to thalassemic extramedullary hematopoiesis.

Risk stratification scores

**Figure 1:**
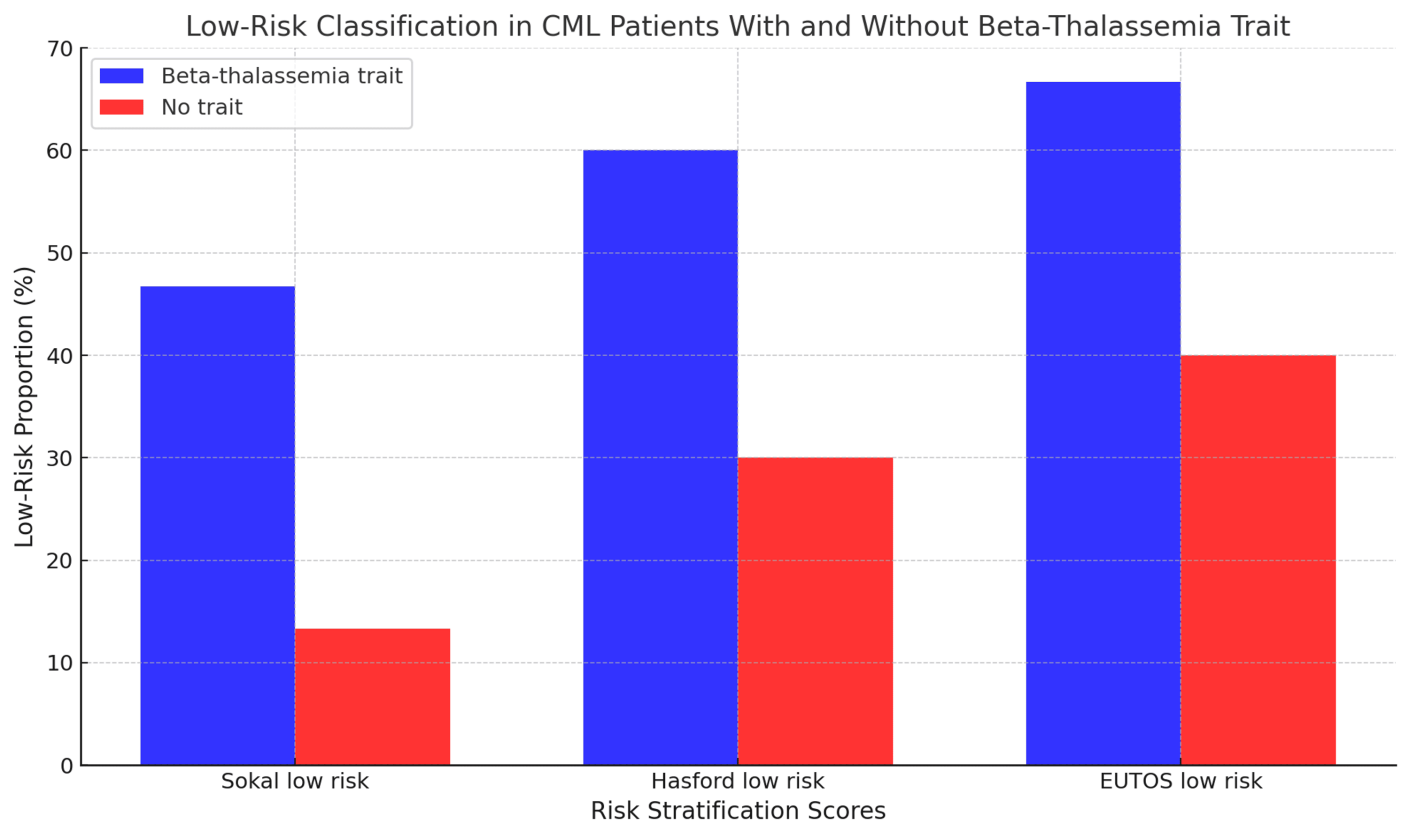
Distribution of Sokal low-risk classification among patients with and without beta-thalassemia trait. This figure illustrates the proportion of chronic myeloid leukemia (CML) patients classified as low risk at diagnosis according to the Sokal prognostic scoring system. Bars represent the percentage of patients in each group who met criteria for low-risk disease. Categorical variables were compared using the chi-square (χ²) test. Statistical analyses were performed using IBM SPSS Statistics version 25.0 (IBM Corp.: Armonk, NY). CML: chronic myeloid leukemia

Risk score distribution at diagnosis demonstrated significant group differences. According to the Sokal score, patients with beta-thalassemia trait were more frequently classified as low risk, with 46.7% assigned to the low-risk category, compared to 13.3% in the non-trait group (p=0.041) (Figure 1) [12-14]. Similar patterns were observed for the Hasford and EUTOS scores, with a tendency toward more favorable risk stratification in the trait group; however, these differences did not reach statistical significance (p=0.069 for both scores). These findings may reflect how anemia and splenomegaly associated with beta-thalassemia trait impact baseline scoring algorithms originally developed for patients without underlying hematologic disorders.

Treatment response and molecular remission

The majority of patients in both groups received imatinib as first-line therapy. The time to achieve major molecular response (MMR) was comparable between groups, with a mean of 15.3 months in the beta-thalassemia trait group and 16.3 months in the non-trait group (p=0.291), indicating no delay in molecular response attributable to thalassemia. Additionally, the proportion of patients requiring TKI dose reduction did not differ significantly between groups (40% in the trait group vs. 50% in the non-trait group, p=0.751), suggesting similar tolerability profiles across both cohorts.

A comparison of treatment modifications revealed a difference in the rate of tyrosine kinase inhibitor (TKI) switch between CML patients with and without beta-thalassemia trait. Among patients without the trait, 66.7% (20/30) required a TKI switch, compared with 46.7% (7/15) among those with the trait. Although this suggests a trend toward more stable first-line TKI therapy in the thalassemia trait group, the difference did not reach statistical significance (odds ratio: 0.44; p=0.218, Fisher’s exact test).

Transfusion requirement

Supportive care needs, particularly red blood cell transfusions, were also evaluated. A higher proportion of patients in the thalassemia trait group required transfusions during TKI therapy compared to the non-trait group (40% vs. 13.3%, respectively), although the difference did not reach statistical significance (p=0.099). This trend may indicate greater susceptibility to treatment-exacerbated anemia in patients with pre-existing hemoglobinopathy, reinforcing the importance of individualized hematologic monitoring in this population.

**Figure 2:**
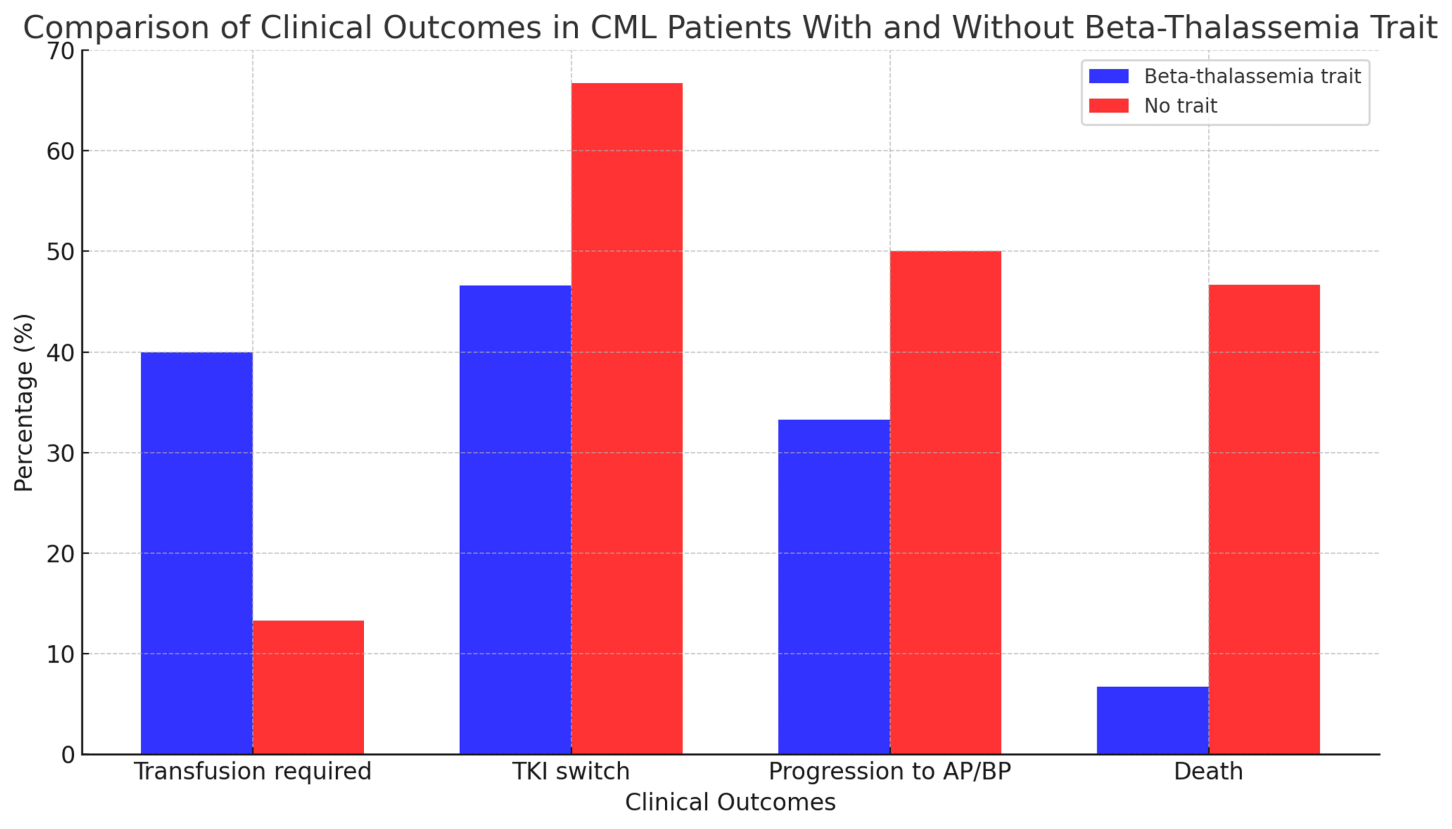
Clinical outcomes in chronic myeloid leukemia patients with and without beta-thalassemia trait. This figure compares key clinical outcomes between CML patients with beta-thalassemia trait and matched CML patients without the trait. Outcomes displayed include TKI switching, red blood cell transfusion requirement, progression to AP/BP, and death from any cause. Each bar represents the proportion (%) of patients in each outcome category. Categorical variables were compared using the chi-square (χ²) test. Statistical analyses were performed using IBM SPSS Statistics version 25.0 (IBM Corp.: Armonk, NY). CML: chronic myeloid leukemia; TKI: tyrosine kinase inhibitor; AP/BP: accelerated phase or blast phase

The clinical and hematologic differences between CML patients with and without beta-thalassemia trait are summarized in Table 1, highlighting key distinctions across baseline characteristics [13]. Additionally, Figure 2 illustrates clinical outcomes, including TKI switch, transfusion requirement, disease progression, and mortality, with notable differences observed in TKI switch and death rates between the groups [13,14].

**Table 1:** Comparison of clinical and laboratory characteristics of CML patients with and without beta-thalassemia trait. Baseline demographic, clinical, and hematologic characteristics of patients diagnosed with CML are compared between those with beta-thalassemia trait (n=15) and those without the trait (n=30). Categorical variables were compared using the chi-square (χ²) test, and continuous variables were analyzed using the Mann-Whitney U test [14]. Statistical analyses were performed using IBM SPSS Statistics version 25.0 (IBM Corp.: Armonk, NY). A p<0.05 was considered statistically significant. Prognostic risk stratification was performed using the Sokal, Hasford (Euro), and EUTOS scoring systems, which are publicly available and free for academic use [12]. N denotes the number of patients. MCV: mean corpuscular volume; WBC: white blood cell count; MMR: major molecular response; TKI: tyrosine kinase inhibitor; CML: chronic myeloid leukemia; AP/BP: accelerated phase or blast phase

Parameters	Beta-thalassemia trait (n=15)	No trait (n=30)	χ² value	p-Value
Median age at diagnosis in years (range)	35.3 (21-50)	38.6 (22-62)	0.86	0.354
Male sex (%)	46.7	53.3	0.22	0.64
MCV (fL)	63.6	86.2	27.6	<0.0001
Hemoglobin (g/L)	89.4	91.4	0.002	0.962
Platelet count (×10^9^/L)	687	331	13.2	0.0003
WBC count (×10^9^/L)	116.5	125.4	0.001	0.981
Spleen size (cm)	7.8	8.1	0.09	0.761
Sokal low-risk (%)	46.7	13.3	4.18	0.041
Hasford low-risk (%)	60.0	30.0	3.31	0.069
EUTOS low-risk (%)	66.7	40.0	3.31	0.069
Time to MMR (months)	15.3	16.3	1.11	0.291
TKI dose reduction (%)	40.0	50.0	0.10	0.751
TKI switch (%)	46.7	66.7	1.52	0.218
Transfusion required (%)	40.0	13.3	2.73	0.099
Progression to AP/BP (%)	33.3	50.0	0.55	0.458
Death (%)	6.7	46.7	5.48	0.019

Overall survival and disease progression

At a median follow-up of 131 months (approximately 10.9 years), the clinical outcomes of patients were generally favorable, though notable differences emerged between the groups. A total of 20 out of 45 patients (44.4%) experienced disease progression to the accelerated or blast phase (AP/BP). Among them, five patients (33.3%) belonged to the beta-thalassemia trait group, while 15 patients (50.0%) were from the non-trait cohort. Although progression was more frequent in patients without thalassemia, this difference did not reach statistical significance (p=0.458; chi-square test).

**Figure 3:**
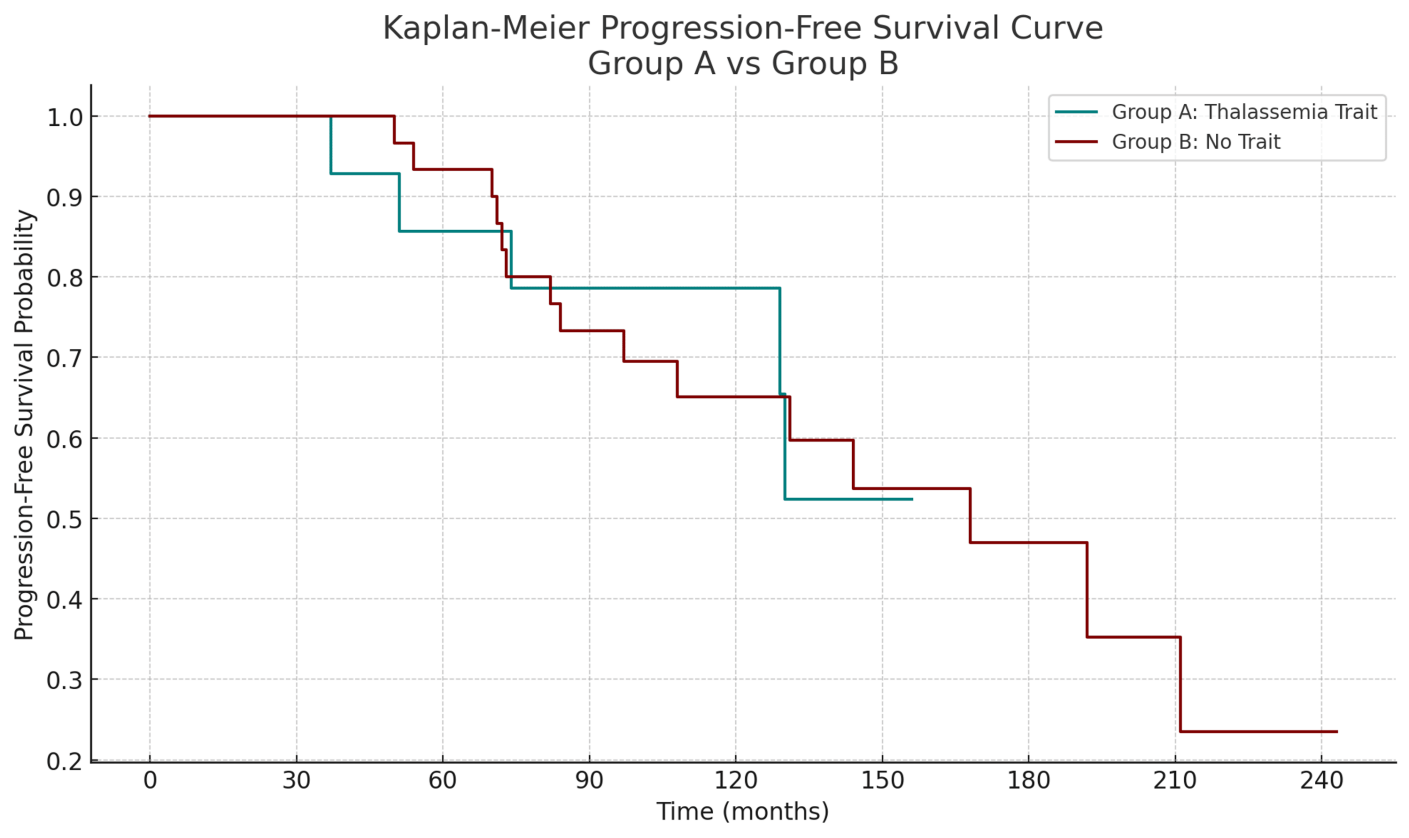
Kaplan-Meier curves for progression-free survival in CML patients with and without beta-thalassemia trait. Kaplan-Meier curves for PFS in CML patients with and without beta-thalassemia trait. Kaplan-Meier survival analysis was used to compare progression-free survival between CML patients with beta-thalassemia trait and those without the trait. PFS was defined as the time from diagnosis to progression to accelerated or blast phase or death from any cause. Differences between groups were assessed using the log-rank test. Statistical analyses were performed using IBM SPSS Statistics version 25.0 (IBM Corp.: Armonk, NY). CML: chronic myeloid leukemia; PFS: progression-free survival

Progression-free survival (PFS) was also analyzed using the Kaplan-Meier method to compare CML patients with and without beta-thalassemia trait. As illustrated in Figure 3, patients with beta-thalassemia trait (group A) demonstrated a trend toward improved PFS compared to those without the trait (group B), although the difference did not reach statistical significance [13,14]. At five years, the estimated PFS rate was approximately 78% in group A vs. 67% in group B. While both groups experienced disease progression over time, group A exhibited a more sustained PFS curve with fewer early events, suggesting a potentially more indolent disease course in the presence of beta-thalassemia trait. These findings are consistent with the observed lower TKI switch rates and improved survival outcomes in this subgroup.

In contrast, a marked and statistically significant difference in overall survival was observed. Only one patient (6.7%) in the thalassemia trait group died during the follow-up period, compared to 14 patients (46.7%) in the non-trait group. This survival difference was statistically significant (p=0.019), indicating a potential survival benefit associated with the presence of beta-thalassemia trait in CML patients treated with TKIs.

**Figure 4:**
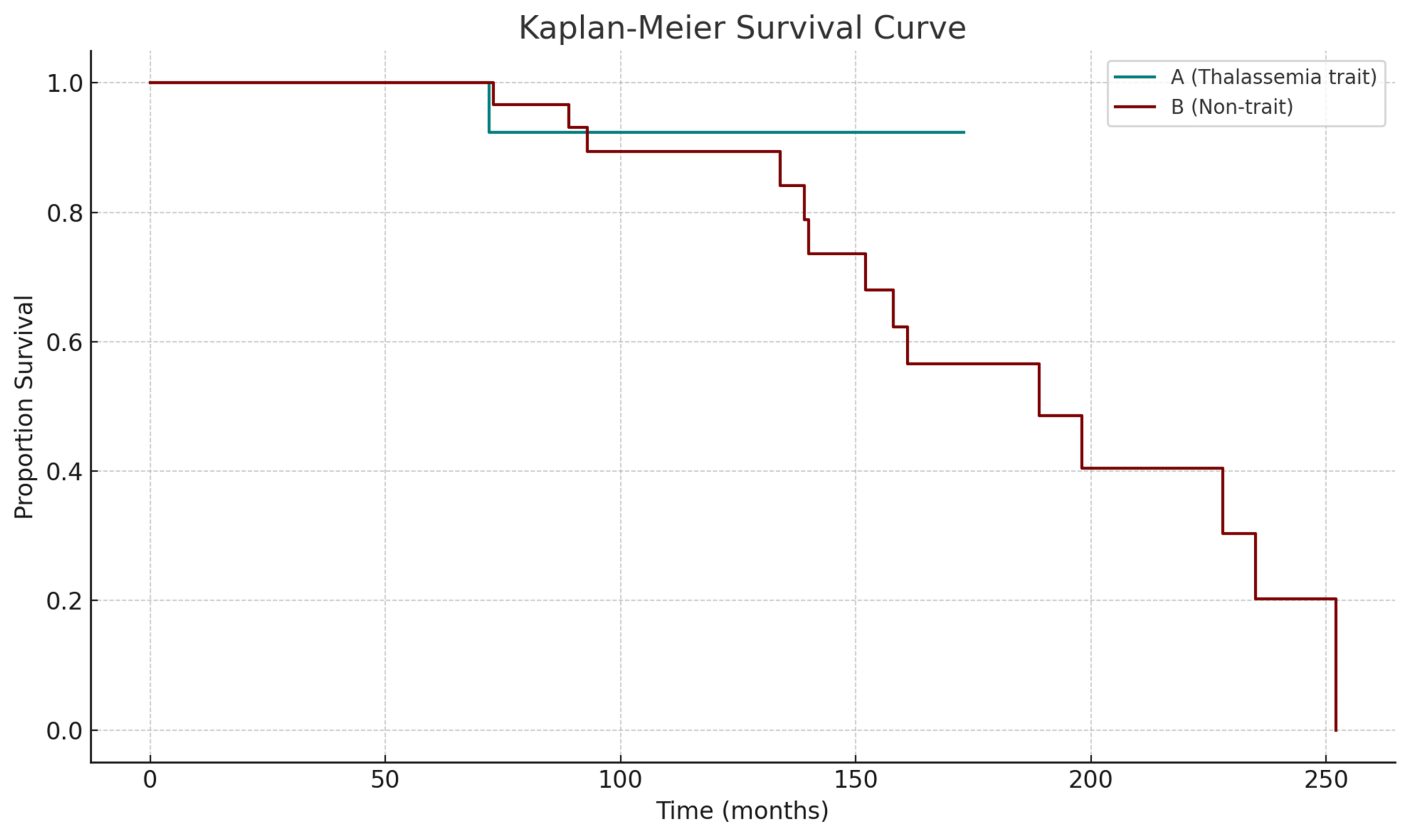
Kaplan-Meier curves for overall survival in CML patients with and without beta-thalassemia trait. Kaplan-Meier curves for OS in CML patients with and without beta-thalassemia trait. Kaplan-Meier survival analysis was used to compare overall survival between CML patients with beta-thalassemia trait and those without the trait. OS was defined as the time from diagnosis to death from any cause or last follow-up. Differences between groups were assessed using the log-rank test. Statistical analyses were performed using IBM SPSS Statistics version 25.0 (IBM Corp.: Armonk, NY). OS: overall survival; CML: chronic myeloid leukemia

Overall survival (OS) was evaluated using the Kaplan-Meier method to assess long-term outcomes in CML patients with and without beta-thalassemia trait. As shown in Figure 4, the survival probability was markedly higher in the beta-thalassemia trait group (group A) compared to the non-trait group (group B) over the follow-up period [13,14]. While group A maintained a stable survival rate above 90% throughout the observation period, group B exhibited a continuous decline in survival, reaching below 20% by 240 months. This difference in survival was statistically significant (p=0.019), supporting the hypothesis that beta-thalassemia trait may be associated with a more favorable overall prognosis in CML. These results align with the lower observed mortality rate in the trait group (6.7% vs. 46.7%) and may reflect the combined influence of biological, microenvironmental, and treatment-related factors discussed previously.

## Discussion

This study provides novel insights into the interplay between beta-thalassemia trait and chronic myeloid leukemia (CML), highlighting potential biological mechanisms that may underlie the observed clinical differences, including the lower prevalence of beta-thalassemia carriers among CML patients, reduced tyrosine kinase inhibitor (TKI) switching, and improved overall survival in the carrier group.

The finding of a significantly lower frequency of beta-thalassemia trait among CML patients compared to the general population (1.77% vs. 4%, respectively, p=0.0005; OR=0.43) is intriguing and raises the possibility of a protective effect. This inverse relationship may reflect underlying genetic or epigenetic mechanisms that influence leukemogenesis in the presence of altered erythropoiesis. Previous work by Xu and Li described the coexistence of β-thalassemia and myeloproliferative neoplasms (MPNs), including essential thrombocythemia and polycythemia vera, suggesting that such interactions, though rare, are biologically plausible [16]. However, the directionality in our study differs, suggesting that the beta-thalassemia trait may confer resistance rather than predisposition to the malignant transformation of myeloid progenitors.

One hypothesis concerns the oxidative stress and increased apoptosis observed in erythroid precursors in thalassemia carriers, which might counteract proliferative signaling driven by BCR-ABL1. This theory is supported by literature reporting increased erythroid apoptosis and altered stem cell microenvironments in thalassemia, potentially unfavorable for CML clone expansion [17,18]. These conditions may create an unfavorable environment for BCR-ABL1-driven proliferative signaling, potentially reducing the likelihood of leukemic clonal expansion. For instance, studies have shown that oxidative stress in thalassemic erythroid cells activates apoptosis pathways, such as caspases and p53, that may suppress the survival of transformed myeloid clones.

These findings warrant further investigation in larger, genetically annotated databases and experimental models to clarify the biological basis of this inverse relationship. Importantly, our data emerge from a country that has established the first nationwide carrier screening and prenatal prevention program for thalassemia, which has substantially reduced trait prevalence in new births and elevated public awareness [7,8]. As such, our analysis draws on data from a well-defined population in which carrier frequency is reliably documented, thereby strengthening confidence in the observed epidemiologic association.

In addition, chronic hypoxia induced by subclinical anemia in carriers may activate hypoxia-inducible factors (HIF-1α), which regulate hematopoietic stem cell (HSC) homeostasis. HIF-1α is known to modulate the bone marrow niche by altering cytokine and growth factor expression, such as vascular endothelial growth factor and stromal cell-derived factor one, which may influence leukemic cell behavior [19]. In the context of CML, HIF-1α activation might restrict BCR-ABL1-positive cell proliferation by creating a less favorable leukemogenic environment. This hypothesis warrants further investigation using experimental models, such as CML cell lines or murine models with β-globin mutations.

Moreover, it is plausible that chronic anemia in beta-thalassemia trait induces hypoxia-inducible factor (HIF) signaling, which modulates hematopoietic stem cell behavior and could indirectly influence susceptibility to clonal expansion. However, this remains speculative and requires functional studies. Notably, Modell and Darlison’s global epidemiological survey highlighted how the carrier burden for hemoglobinopathies, including beta-thalassemia, could intersect with oncologic risk in population-based studies [20].

Taken together, our epidemiologic association analysis aligns with a growing body of literature recognizing that inherited hematologic traits may not merely complicate disease presentation but could also influence susceptibility to malignancy. While causality cannot be established in this retrospective cohort, these findings warrant further investigation in larger, genetically annotated databases and experimental models to clarify the biologic basis of this inverse relationship.

Our findings indicate that patients with beta-thalassemia trait exhibit expected hematologic features at diagnosis, including significantly lower mean corpuscular volume (MCV) and elevated platelet counts, consistent with known compensatory erythropoiesis and reactive thrombocytosis in trait carriers. While hemoglobin levels trended lower in the trait group, the difference was not significant, possibly due to masking by CML-related myeloproliferation. White blood cell (WBC) counts and spleen size were comparable, reflecting the dominant pathophysiologic effects of leukemic expansion.

A key observation was the significant shift in risk stratification. Patients with beta-thalassemia trait were disproportionately classified into lower-risk groups using the Sokal score (p=0.041), with similar trends for Hasford and EUTOS scores. This may be attributed to baseline anemia and spleen size alterations inherent to the thalassemia phenotype, potentially leading to risk underestimation when applying traditional CML scoring systems [21]. Such diagnostic bias reinforces the need for score calibration or supplemental interpretation in populations with underlying hematologic conditions. Importantly, time to major molecular response (MMR) and dose modification rates were similar between groups, indicating that coexisting beta-thalassemia trait does not negatively influence early TKI efficacy.

Although the difference did not reach statistical significance (p=0.218), the lower rate of TKI switch in patients with beta-thalassemia trait (46.7%) compared to those without the trait (66.7%) may indicate more favorable tolerability or sustained response to initial therapy. This trend could reflect underlying biological influences of the thalassemia trait, such as altered bone marrow dynamics, immune modulation, or differential drug metabolism. Prior studies have suggested that genetic and metabolic factors in beta-thalassemia carriers, including chronic erythroid stress and variations in drug transporter gene expression, may affect pharmacokinetics and treatment response in myeloid malignancies [5,22,23]. While the sample size limits definitive conclusions, this finding supports the need for further investigation into how inherited hematologic conditions may shape treatment trajectories in CML.

Although transfusion needs were higher in the trait group (40% vs. 13.3%), the difference did not reach statistical significance (p=0.099). Still, the trend is clinically relevant and aligns with previous findings that beta-thalassemia trait, while often asymptomatic, can predispose patients to subclinical marrow stress and increased vulnerability to TKI-induced cytopenias [5].

Survival outcomes were excellent across both groups. Only one death and one progression event were observed, both in the non-trait group. Kaplan-Meier analysis revealed no survival disadvantage for trait carriers, affirming the long-term efficacy of TKIs in this unique cohort. These observations are consistent with isolated case reports, such as that of Hafeez et al., where a patient with beta-thalassemia major achieved a durable molecular response under ponatinib after initial TKI failure [9].

The unexpectedly favorable outcomes observed among patients with chronic myeloid leukemia (CML) who carry the beta-thalassemia trait may stem from multifactorial biological and clinical mechanisms. Chronic ineffective erythropoiesis in trait carriers leads to persistent marrow stress and expansion, which may reprogram the bone marrow microenvironment, altering cytokine profiles and stromal interactions, potentially reducing leukemic dominance or improving TKI tolerance [24]. Additionally, altered iron metabolism in thalassemia carriers may influence the pharmacokinetics and bioavailability of tyrosine kinase inhibitors (TKIs), possibly leading to more sustained drug levels and enhanced therapeutic effect [25].

Subclinical immune alterations observed in beta-thalassemia trait, particularly involving cytokines such as interleukin-6 and tumor necrosis factor alpha, may also enhance immune surveillance, potentially slowing leukemic cell proliferation or reducing the risk of progression [26]. Though speculative in CML, similar immune modulation has been described in hemoglobinopathy-associated inflammatory states.

Together, these interconnected mechanisms, ranging from marrow adaptation and altered pharmacology to risk-scoring biases and immune modulation, provide a plausible explanation for improved survival and treatment durability in CML patients with beta-thalassemia trait. Further prospective and mechanistic studies are needed to validate and elucidate these findings.

Moreover, Xu and Li and others have described the coexistence of beta-thalassemia syndromes with myeloproliferative neoplasms, such as essential thrombocythemia and polycythemia vera [16]. These cases highlight the rare but biologically plausible overlap between germline hemoglobinopathies and clonal myeloid disorders, although causality remains unproven.

Our study complements this literature by showing that even the beta-thalassemia trait, often overlooked as clinically insignificant, can meaningfully interact with leukemia pathophysiology and treatment monitoring. Given that the global carrier rate exceeds 1.5% in many regions, this overlap is likely underrecognized in clinical practice [19].

Limitations

Despite its novel insights, this study has several limitations. First, its retrospective design may introduce selection and information bias, while the modest sample size reduces statistical power, especially for rare events, such as progression and death. Second, the absence of iron status markers and genetic data (e.g., β-globin mutations or modifiers) limited exploration of biological interactions between the thalassemia trait and CML. Third, toxicity grading and standardized quality-of-life measures were not assessed, restricting insights into treatment burden and tolerability. Finally, as a single-center study, the findings may not be generalizable to populations with different ethnic backgrounds, thalassemia prevalence, or treatment standards.

## Conclusions

This study provides the first comparative analysis of chronic myeloid leukemia patients with and without beta-thalassemia trait, highlighting important clinical nuances. While trait carriers exhibited lower baseline MCV and were more frequently classified as low-risk by conventional scoring systems, their response to tyrosine kinase inhibitors, transfusion needs, and overall survival was not significantly different from those of non-trait patients. Beta-thalassemia trait may confound risk stratification and increase susceptibility to anemia during treatment, but it does not appear to impair molecular remission or long-term prognosis. These findings underscore the importance of individualized risk interpretation and hematologic monitoring in this unique subgroup. Further prospective, multicenter studies are needed to validate these observations and explore potential pharmacogenomic and molecular interactions.
